# *Fucus vesiculosus*-Rich Extracts as Potential Functional Food Ingredients: A Holistic Extraction Approach

**DOI:** 10.3390/foods13040540

**Published:** 2024-02-09

**Authors:** Ana R. Circuncisão, Sónia S. Ferreira, Artur M. S. Silva, Manuel A. Coimbra, Susana M. Cardoso

**Affiliations:** LAQV-REQUIMTE, Department of Chemistry, University of Aveiro, 3810-193 Aveiro, Portugal; anarcircuncisao@ua.pt (A.R.C.); soniasferreira@ua.pt (S.S.F.); artur.silva@ua.pt (A.M.S.S.); mac@ua.pt (M.A.C.)

**Keywords:** brown macroalgae, *Fucus vesiculosus*, functional food ingredients, sustainable extraction, phlorotannins, fucoxanthin, alginates, fucoidans, laminarans

## Abstract

Brown macroalgae are rich sources of nutrients and health-promoting compounds. Nevertheless, their consumption is still limited by their strong organoleptic characteristics, thus requiring the development of extraction strategies to profit from their nutritional value. To fulfil this, two sequential extraction approaches were developed, differing in the solvent used in the first extraction step, water in approach 1 or food-grade ethanol in approach 2, to obtain economic and affordable extracts rich in specific compounds from *Fucus vesiculosus.* The use of water in the first step of extraction allowed us to recover water-soluble phlorotannins, laminarans and mannuronic-rich alginates, making the subsequent 70% ethanol extract richest in fucoxanthin (0.07% algae DW), and the hot water fractions purest in fucoidans and alginates with a lower mannuronic-to-guluronic (M/G) ratio (2.91). Conversely, when beginning extraction procedures with 96% ethanol, the recovered yields of phlorotannins increased (0.43 g PGE/100 g algae DW), but there was a concomitant seven-fold decrease in the recovery of fucoxanthin in the subsequent 70% ethanol extract. This approach also led to less pure hot water fractions containing fucoidans, laminarans and alginates with a higher M/G ratio (5.50). Overall, this work unveiled the potential of the first extraction steps in sustainable and holistic cascade strategies to modulate the composition of food-grade extracts, creating prospects of their application as tailored functional ingredients in food products.

## 1. Introduction

*Fucus vesiculosus* L. (Fucaceae), traditionally named bladder wrack, has shown great potential for valorization [1]. Like other brown macroalgae, *F. vesiculosus* is recognized for its abundance of specific phenolic compounds, named phlorotannins (PTs), of the orange-colored pigment fucoxanthin, of minerals like iodine and calcium, and of bioactive polysaccharides, namely fucoidans, laminarans and alginates. Notably, all these compounds have been consistently shown to possess important biological activities [1,2,3]. Phlorotannins are highly hydrophilic molecules consisting of dehydro-oligomers or dehydro-polymers formed through the C–C and/or C–O–C oxidative coupling of phloroglucinol (1,3,5-trihydroxybenzene) monomeric units [1,4]. They can be found with molecular weights ranging from 126 Da to 650 kDa and in different assemblages, which increases their structural complexity [4]. In *Fucus* sp., PTs can reach up to 12% of the dry weight (DW), with fucols and fucophlorethols being the most common representative class in *F. vesiculosus* [4,5]. As phenolics, PTs have been found to be potent anti-oxidant and anti-inflammatory agents [1,6]. Fucoxanthin is a low-polar photosynthetic xanthophyll belonging to the tetraterpenoid family with a structure consisting of an unusual allenic bond, a 5,6-monoepoxide and nine conjugated double bonds. In *Fucus* spp., this pigment has been reported to reach 0.4% DW [7]. Fucoxanthin is recognized for its anti-obesity and anti-diabetic effects [8,9].

The polysaccharides of brown macroalgae include both structural carbohydrates, like alginates and fucoidans (fucose-containing sulphated polysaccharides, FCSPs), and storage carbohydrates such as laminarans [2]. Alginates are linear acidic polysaccharides composed of β-1,4-d-mannuronic acid (M) and α-1,4-l-guluronic acid (G), arranged either in heteropolymeric (MG) and/or homopolymeric (M or G) blocks. Alginates can reach 59% of the DW of *F. vesiculosus*, and, due to their low toxicity and biocompatibility, they have been applied as potential gelling, stabilizing, and thickening agents in the food industry [10]. Regarding fucoidans, they are water-soluble and heterogenous polysaccharides composed mainly of fucose and sulphate, although other monosaccharides (mannose, galactose, glucose, xylose and uronic acids) or even acetyl groups may be present [3]. Fucoidans can reach 26% of the DW of *F. vesiculosus*, with a molecular weight of up to 1600 kDa [1,11]. These polysaccharides have received more attention due to their low toxicity and well-established anticoagulant and immunomodulatory effects [12,13]. Laminarans are polysaccharides composed of a linear backbone of β-1,3-d-glucopyranose residues with some random 6-O-branching points. Compared to other brown polysaccharides, they are low-molecular-weight compounds (up to 5 kDa). In *F. vesiculosus*, these were found to reach 7% DW [1,14]. Since laminarans do not form viscous solutions nor gels, their commercial interest has emerged from their bioactivities, including their anti-oxidant, anti-microbial and immunomodulatory effects [5,15].

Due to the richness of its bioactive compounds, *F. vesiculosus* has been suggested as a valuable food ingredient. The inclusion of seaweeds into food products has been proposed as a strategy to be used by the food industry to profit from their nutritional and potential health-benefits [5]. However, seaweeds’ strong taste and perceived flavor are critical factors limiting the proportion of algae that can be used as food ingredients, which may result in a drawback in fully reaching their well-known profitability. In this regard, the targeted extraction of seaweeds’ valuable compounds can overcome these limitations, preferably at a low cost and using environmentally friendly practices.

Sequential extraction has been pointed out as a promising strategy, since it may allow biomass to be continuously extracted via the utilization of several solvents with different polarities, following the expected targets. Notably, the development of seaweeds’ biorefinery processes able to integrate sequential extraction to produce multiple products is very limited, compared with the extensive research conducted on single-product extraction. In fact, few studies cover the sequential extraction of bioactive compounds from brown seaweeds [16,17,18,19]. Moreover, from our perspective, most of them at some point show discarded potential fractions or overly purified processes, which unfortunately make any chance of industrial scaling-up difficult. For instance, Zhang et al. [20] developed a fully operational *F. vesiculosus* biorefinery system at the lab and pilot scale, but they only focused on the production of food-grade fucoidan and laminaran, overlooking other valuable compounds (e.g., alginates and phlorotannins).

Considering that biorefining of brown seaweeds is still a very immature field, the present study aimed to obtain economic, affordable extracts rich in specific target compounds, including phlorotannins, fucoxanthin, fucoidans, laminarans and alginates, from a commercially important European brown macroalga, namely *F. vesiculosus*, following two different holistic, sequential, and low-chemical extraction approaches. The obtained food-grade extracts and/or fractions and the respective final residual biomass were characterized. The impacts of the sequential extraction approaches on the composition of the extracts and/or fractions were also discussed, taking into consideration their potential application as food ingredients. To the best of our knowledge, this is the first report on the production of several high-value-added compounds from *F. vesiculosus* via a holistic and biorefinery process.

## 2. Materials and Methods

### 2.1. Samples and Reagents

Grounded brown *F. vesiculosus* macroalga collected in 2021 were purchased from Algaplus, Lda (https://www.algaplus.pt/, accessed on 10 July 2023), a local company specialized in the production of edible seaweeds in a land-based integrated multi-trophic aquaculture (IMTA) system and their commercialization in the food and cosmetic markets. Glacial acetic acid, hydrochloric acid (HCl), formic acid, sulphuric acid (H_2_SO_4_), sodium borohydride (NaBH_4_), dichloromethane, HPLC-grade ethanol, acetonitrile, acetone, and methanol were purchased from Fisher Chemical^TM^, Middlesex County, MA, USA. 2,5-dimethoxybenzaldehyde (DMBA), phloroglucinol, Folin–Ciocalteu reagent, 2-deoxyglucose, 1-methylimidazole and potassium sulphate were purchased from Sigma-Aldrich, Madrid, Spain. Food-grade ethanol (96%, *v*/*v*) and sodium carbonate were purchased from PanReac AppliChem, Barcelona, Spain. Calcium chloride was purchased from Chemlab—Analytical, Zedelgem, Belgium. Acetic anhydride was purchased at Carlo Erba Reagents, Cornaredo, Milano, Italy. 3-phenylphenol, barium chloride (BaCl_2_) and NaOH solution (50%) for HPAEC were purchased from MERCK. Sodium acetate for HPAEC was purchased from Thermo Scientific^TM^ Dionex^TM^ AAA-Direct Reagents, Waltham, MA, USA. The standards fucoxanthin, galacturonic acid, glucuronic acid, mannuronic acid and guluronic acid were acquired from Sigma-Aldrich, Madrid, Spain. All reagents were of analytical grade or of the highest available purity.

### 2.2. Preparation of Fucus vesiculosus Extracts

**Figure 1:**
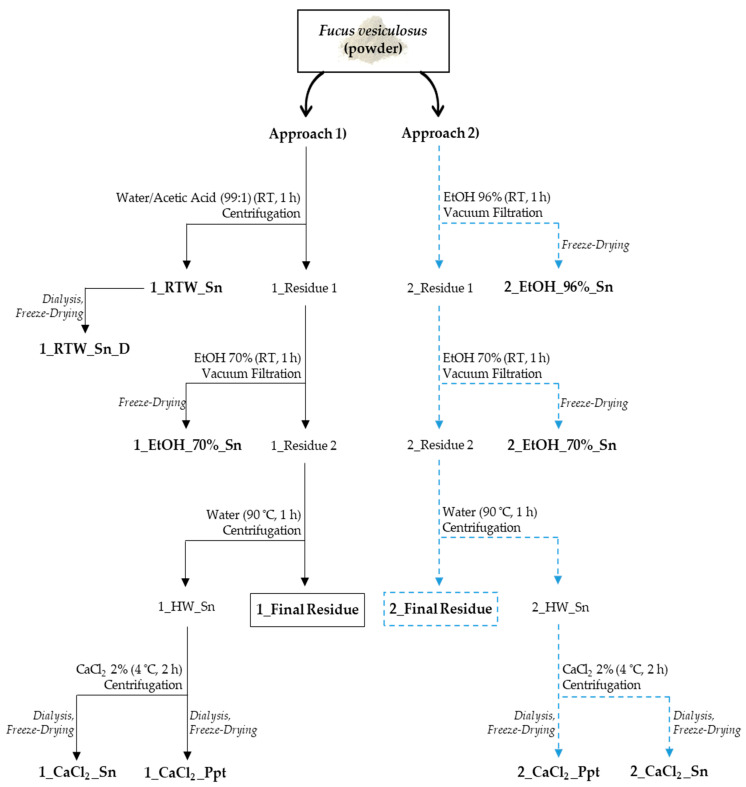
Schematic representation of the first and second holistic extraction approaches developed for brown *F. vesiculosus* macroalga. The fractions that were studied in this work are marked in bold.

Based on preliminary tests, water and 96% ethanol yielded the highest recovery of total phenolics; therefore, they were selected to be used as the first steps in two different extraction strategy approaches. The schematic representation of the holistic sequential extraction procedure of phlorotannins, fucoxanthin and polysaccharides developed for *F. vesiculosus* is shown in Figure 1. In approach 1, *F. vesiculosus* powder was added to water/acetic acid (99/1, *v*/*v*) solution at a ratio of 1:70 (*w*/*v*) and extracted at room temperature (RT) for 1 h, under constant stirring. The resulting suspension was centrifuged (6000 rpm, 20 min, 4 °C), and the obtained residue was sequentially extracted as previously carried out, but using an ethanol/water (70/30, *v*/*v*) solution at a ratio of 1:30 (*w*/*v*). This suspension was vacuum-filtered, and the resulting soluble fraction (1_EtOH_70%_Sn) and the soluble fraction from RT water extraction (1_RTW_Sn) were separately concentrated on a rotary evaporator at 30 °C and freeze-dried. In addition, to study polysaccharides from 1_RTW_Sn in more detail, this extract was dialyzed (12–14 kDa cut-off membrane (MWCO), Medicell) at 4 °C. Following that, the dialyzed water extract (1_RTW_Sn_D) was concentrated on a rotary evaporator at 30 °C and freeze-dried for further analysis.

On the other hand, in approach 2, the *F. vesiculosus* powder was first submitted to extraction with ethanol/water (96/4, *v*/*v*) at a ratio of 1:70 (*w*/*v*) at RT for 1 h, under constant stirring. The obtained suspension was vacuum-filtered, and the resulting residue was sequentially extracted and vacuum-filtered following a similar procedure to that used before, but using ethanol/water (70/30, *v*/*v*) at a ratio of 1:20 (*w*/*v*). The ethanolic soluble fractions (2_EtOH_96%_Sn and 2_EtOH_70%_Sn) were separately concentrated on a rotary evaporator at 30 °C and freeze-dried. Afterwards, in order to recover polysaccharides, residues from both approaches (1_Residue 2 and 2_Residue 2) were successively extracted with water at a ratio of 1:100 (*w*/*v*), at 90 °C for 1 h, under constant stirring. The obtained suspensions were separated via centrifugation, and the resulting hot-water-insoluble fractions (1_Final Residue and 2_Final Residue) were freeze-dried. In order to separate alginates, the volume of corresponding hot-water-soluble fractions (1_HW_Sn and 2_HW_Sn) was measured and calcium chloride (CaCl_2_) was added until it reached a concentration of 2% (*w*/*v*). This suspension was slowly stirred at RT for 15 min followed by 2 h at 4 °C (cold chamber). After centrifugation, the recovered soluble fractions (1_CaCl_2__Sn and 2_CaCl_2__Sn), and the precipitates (1_CaCl_2__Ppt and 2_CaCl_2__Ppt) were extensively dialyzed (12–14 kDa MWCO) at 4 °C in order to remove CaCl_2_ from the samples. Following that, samples were concentrated on a rotary evaporator at 30 °C and freeze-dried for further analysis.

### 2.3. Chemical Characterization of F. vesiculosus Powder and/or Extracts

#### 2.3.1. Total Ash and Protein Content

*F. vesiculosus* powder (500 mg) was placed into pre-weighed dry porcelain crucibles and put in a muffle furnace at 700 °C for 6 h, under an air atmosphere. After cooling down, ash content (%) was quantified via gravimetry.

Total protein content was estimated via the determination of elemental nitrogen (N) content using thermal conductivity with a Truspec 630-200-200 CHNS analyzer from LECO (St Joseph, MI, USA). The protein content was calculated using a nitrogen-to-protein conversion factor of 5, as previously suggested by Angell et al. [21] for macroalga biomass.

#### 2.3.2. Total Phlorotannins and Phenolics Content

Total phlorotannins were quantified in accordance with the 2,4-dimethoxybenzaldehyde (DMBA) colorimetric method using phloroglucinol as the standard (6–100 µg/mL), as described by Ferreira et al. [22]. Briefly, 50 µL of selected samples was added to 250 µL of a work solution, at a ratio of 1:1 (*v*/*v*), composed of DMBA (2%; *w*/*v*) and HCl (6%, *w*/*v*) prepared in glacial acetic acid. After 60 min of incubation, absorbance was recorded at 515 nm. The quantification of total phenolic compounds (TPCs) was carried out following the Folin–Ciocalteu general methodology described by Silva et al. [23], with minor modifications. Briefly, 60 µL of deionized water was added into a 96-well plate followed by the addition of 15 µL of Folin–Ciocalteu reagent and 15 µL of the selected samples/standard, and incubated 5 min at room temperature. After that, 150 µL of 3.5% (*w*/*v*) sodium carbonate solution was added and incubated for 60 min at 30 °C. Absorbance was recorded at 750 nm, and phloroglucinol (0.025–0.30 mg/mL) was also used as a standard. The total phlorotannin and total phenolic contents were expressed as g of phloroglucinol equivalents/100 g sample (g PGE/100 g extract).

#### 2.3.3. Total Fucoxanthin Content

Fucoxanthin content was determined via ultra-high-performance liquid chromatography (UHPLC) using a photodiode-array detector (DAD), as described by Silva et al. [23], with some modifications. Briefly, each sample was properly diluted in ethanol/water (80/20) and filtered through a nylon filter measuring 0.22 µm (Whatman^TM^, Buckinghamshire, UK) into an amber vial, and stored at −20 °C until injection. UHPLC-DAD analysis was performed on an Ultimate 3000 (Dionex Co., San Jose, CA, USA) apparatus equipped with a quaternary pump, an autosampler, 3000 Diode Array Detector (Dionex Co., San Jose, CA, USA), and an automatic thermostatic column compartment. Separation was achieved by using a Hypersil Gold C18 column (100 mm length; 2.1 mm i.d.) with a particle diameter of 1.9 µm, end-capped from Thermo Scientific (Waltham, MA, USA) and maintained at 30 °C. The mobile phase was composed of 0.1% formic acid in water (Solvent A) and a solution of acetonitrile/methanol (70/30; *v*/*v*) (Solvent B) with a flow rate of 0.200 mL/min in a linear gradient. The solvent gradient started with 15–28% of solvent B over 3.9 min, increasing to 100% in 2.2 min and maintaining this value up to 25 min, followed by a return to the initial conditions, with a total running time of 20 min. Control and data acquisition were carried out with the Thermo Xcalibur Qual Browser data system (Thermo Scientific). The identification of fucoxanthin on samples was performed via a comparison of retention times and absorption spectra at 450 nm with the fucoxanthin standard.

#### 2.3.4. Carbohydrate Analysis

Samples were analyzed for their sugar composition, which was determined as being made up of alditol acetate derivatives, as described by Bastos et al. [24]. Briefly, a pre-hydrolysis step was performed with 72% (*w*/*w*) sulfuric acid for 3 h at room temperature, followed by hydrolysis with 1 M sulfuric acid for 2.5 h at 100 °C. 2-deoxyglucose (1 mg/mL) was used as an internal standard. Monosaccharides were reduced with NaBH_4_ and acetylated with acetic anhydride using methylimidazole as a catalyst. The formed alditol acetate derivatives were analyzed via gas chromatography with a flame ionization detector (GC-FID) (Perkin Elmer Clarus 400, Shelton, CT, USA). Uronic acids (UA) were quantified via the *m*-phenylphenol colorimetric method using α-d-galacturonic acid (10–80 µg/mL) as a standard, and absorbance was measured at 520 nm as described by Bastos et al. [24]. The total carbohydrates were determined via the sum of the amount of individual sugars. Cellulosic glucose was calculated as the difference between the content found with and without pre-hydrolysis with 72% (*w*/*w*) H_2_SO_4_.

#### 2.3.5. Sulphation Degree

Sulphate ester content was determined via turbidimetry using the barium chloride (BaCl_2_) method, as described by Dodgson et al. [25] and Oliveira et al. [26], with minor modifications. The samples were submitted to hydrolysis with 1 mL of 1 M HCl at 105–110 °C for 5 h. Following that, 1.9 mL of 3% (*w*/*v*) trichloroacetic acid and 0.5 mL of BaCl_2_-gelatine reagent (0.5 g of BaCl_2_ in 100 mL of 0.5% (*w*/*v*) gelatine solution) were added to 0.1 mL of the hydrolysate, which was kept at room temperature for 15–20 min. The solution was transferred to quartz microplate wells and analyzed at 360 nm against a reagent blank containing gelatine solution instead of the BaCl_2_–gelatine reagent. The concentration of sulphate esters was determined using K_2_SO_4_ as a standard (0.05 to 2.5 mg/mL).

#### 2.3.6. M/G Ratio

**Figure 2:**
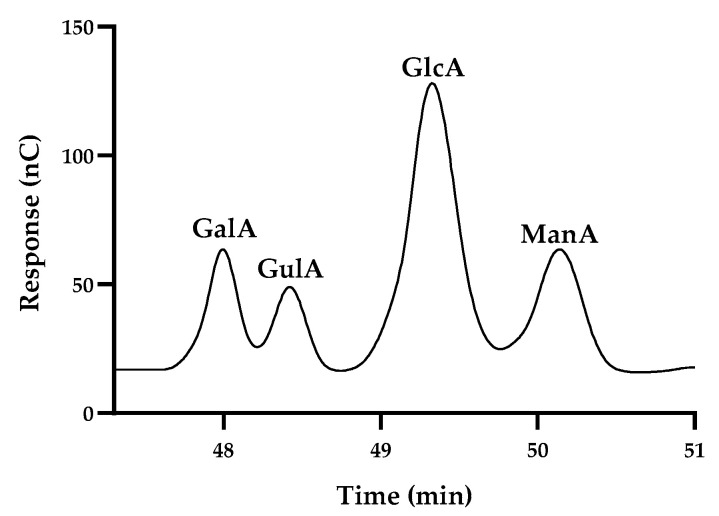
HPAEC-PAD chromatographic profiles of galacturonic (GalA), guluronic (GulA), glucuronic (GlcA) and mannuronic (ManA) acid standards.

The ratio between mannuronic and guluronic acid (M/G) was determined via acid hydrolysis with 200 µL of 72% (*w*/*w*) H_2_SO_4_ for 3 h at room temperature, followed by 1 h at 100 °C in 1 M H_2_SO_4_. After cooling, the samples were diluted in MilliQ water in a proportion of 1:5 (*v*/*v*), filtered through a nylon filter measuring 0.22 µm (Whatman^TM^, Buckinghamshire, UK), and analyzed via high-performance anion exchange chromatography with pulsed amperometric detection (HPAEC-PAD), as reported by Concórdio-Reis et al. [27] and Zhang et al. [28], with some modifications. HPAEC-PAD analysis was carried out on a Dionex ICS-6000 system composed of a DC chromatography oven, a SP pump, and an AS-AP autosampler. Uronic acids were detected using an electrochemical detector in integrated amperometry mode with a AgCl reference electrode and a conventional permanent gold electrode, using Chromeleon 7.3 software (Thermoscientific Dionex, Waltham, MA, USA) and the standard carbohydrate quadrupole waveform recommended for use with CarboPac columns. Separation of compounds was performed using a Dionex CarboPac PA100 guard column (50 mm × 4 mm) and a Dionex CarboPac PA100 analytical column (250 mm × 4 mm) using a gradient elution of 1 mL/min flow rate. The eluents used were MilliQ water (Eluent A), 500 mM sodium hydroxide (NaOH) (Eluent B) and 1 M sodium acetate with 100 mM NaOH (Eluent C). The eluents were prepared using MilliQ water (18 MΩ.cm resistance or higher); 52.8 mL of 50% NaOH solution purchased from MERCK to prepare 2 L of eluent B; 82.0 g of sodium acetate purchased from Thermo Scientific^TM^ Dionex^TM^ AAA-Direct Reagents (Waltham, MA, USA); and 5.28 mL of 50% NaOH solution to prepare 1 L of eluent C. Eluent C was filtered through a 0.22 µm nylon filter and all eluents were used within one week. The temperature of the column and the detector were set at 30 °C and at initial equilibrium with 100% of eluent A. After the injection of 25 µL of the sample onto the column, the carbohydrates were eluted via the following method: an initial step of A:B:C 100:0:0 (*v*/*v*/*v*) for 40 min; a gradient to 82:3:15 from 40 to 45 min; a step of 82:3:15 from 45 to 55 min. After a step of 0:75:25 for 5 min, 100% of eluent B was kept for 10 min to clean impurities, and the eluent proportion was returned to the initial conditions and held for an equilibration time of 15 min. Calibration curves were prepared for galacturonic acid (y = 0.364x in the range 8.0–100 mg/L), guluronic acid (y = 1.725x in the range 8.8–44 mg/L), glucuronic acid (y = 0.906x in the range 1.3–100 mg/L), and mannuronic acid (y = 1.6244x in the range 8.0–80 mg/L). The HPAEC-PAD chromatographic profiles of the four standards can be found in Figure 2.

#### 2.3.7. Statistical Analysis

All experiments were performed with at least three independent assays. Statistical analysis was performed using a trial version of GraphPad Prism version 8.01 software (OriginLab Corporation, Northampton, MA, USA). Data from the M/G ratio and non-cellulosic and cellulosic Glc were analyzed via a one-way ANOVA followed by Tukey’s multiple comparison test. The confidence level was set at 95% with a significance level of *p* < 0.05.

## 3. Results and Discussion

### 3.1. Fucus vesiculosus Composition

**Figure 3:**
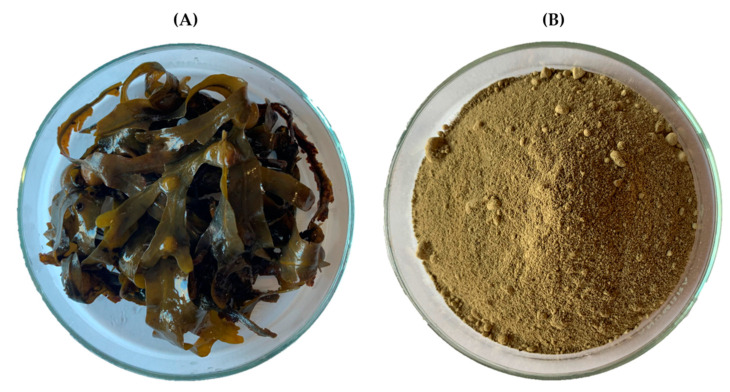
Brown alga *Fucus vesiculosus*. (**A**) Fresh and (**B**) powder.

Brown alga *F. vesiculosus* (Figure 3A) yields a powder with a greenish color (Figure 3B). On a dry-weight (DW) basis, it is composed of 27.8% ash, a level slightly higher than the range between 17.3% and 25.5% DW reported in the literature for the same species [29,30,31,32,33,34]. Ash content is a key indicator of seaweeds’ mineral composition. In the case of brown seaweeds thriving within a marine environment, their mineral composition is recognized not only via the presence of sodium chloride, but also of calcium and sulphates associated with the structure of alginates and sulphated polysaccharides, respectively [1,2]. A higher ash content may be an indication of lower chloride removal during the washing process. In addition, *F. vesiculosus* is composed of 9.6% DW protein (Table 1), consistent with the protein range observed by other authors [1,29,30], although lower levels corresponding to 3.1% DW and 5.8% DW have also been reported [31,32]. Such discrepancies between protein levels may occur due to differences in seasonal and geographic factors [1,35].

The total carbohydrate content comprises 29.4% of the DW of *F. vesiculosus* biomass (Table 1), which is within the range between 17.7 and 35.1% DW [30,31,36]. The total carbohydrates are composed of uronic acids (UA, 33.3 mol%), Fuc (23.5 mol%), Man (21.7 mol%) and Glc (14.4 mol%), with minor amounts of Gal, Xyl and Ara. It was also possible to see the presence of sulphates, accounting for 5.8% of the DW of *F. vesiculosus* biomass (Table 1). Therefore, the carbohydrate composition herein described is consistent with the presence of different polysaccharides in *F. vesiculosus*, as reported for this species [11,37,38] or brown seaweeds in general [1,13,39,40].

### 3.2. Recovery of Phlorotannin- and Fucoxanthin-Rich Extracts

In order to quantify the phlorotannins and fucoxanthin of *F. vesiculosus* powder, which are the compounds responsible for its greenish color, two extraction approaches were developed using food-grade solvents (Figure 1). In approach 1, the alga was extracted with water followed by extraction with 70% aqueous ethanol, both at room temperature. In approach 2, 96% ethanol was used followed by extraction with 70% ethanol. Note that, in general, the most common protocols used for the extraction of phenolics and pigments are based on binary aqueous mixtures with organic solvents, including acetone, ethanol or methanol [4,41].

In approach 1, the water at room temperature (1_RTW_Sn) extracted 38.3% (*w*/*w*) of the *F. vesiculosus* material (Table 1), accounting for 0.36 g of phlorotannins (PTs) expressed as g of phloroglucinol equivalents per 100 g of extract. Subsequent extraction with 70% aqueous ethanol (1_EtOH_70%_Sn) yielded 5.5% (*w*/*w*) of the seaweed material, and this was revealed to contain almost six-fold more PTs than the water extract, thus accounting for 84.9% of the tannins solubilized with both solvents. Regarding the second extraction approach, the 96% ethanol extract (2_EtOH_96%_Sn) was able to recover 18.0% (*w*/*w*) of the *F. vesiculosus* material, while 14.0% was recovered in the subsequent extraction with 70% ethanol (2_EtOH_70%_Sn). The results indicate that 2_EtOH_96%_Sn was the purest extract in PTs, comprising 2.38 g, while 2_EtOH_70%_Sn accounted for 0.63 g, resulting in a total of 3.01 g, which was shown to be 20.6% higher than the total amount of PTs recovered in the first approach (2.39 g). A similar tendency was observed for the TPCs, which were also found to be highest in 2_EtOH_96%_Sn and in the 1_EtOH_70%_Sn, with values corresponding to 8.4 and 2.5 g PGE/100 g extract, respectively (Table 1). In addition, the amount of phlorotannins in the 2_EtOH_96%_Sn extract accounted for 0.43 g PGE/100 g of algae dry weight (DW), making it the richest extract in these compounds compared to the 0.14 g in 1_RTW_Sn, the 0.11 g in 1_EtOH_70%_Sn and the 0.09 g in 2_EtOH_70%_Sn. The concentration of PTs in the 2_EtOH_96%_Sn extract was revealed to be about three-fold lower than those previously reported [42,43]. Such differences may be in part attributed to the use of the Folin–Ciocalteu assay in the previous reports, which often leads to overestimated PTs levels, compared to the more specific 2,4-dimethoxybenzaldehyde (DMBA) assay, which was used in this study for PT quantification. Also, the content of PTs in the 1_RTW_Sn extract was shown to be 10-fold higher than that described in water extracts from *F. vesiculosus* obtained at 25 °C over 1 h at a ratio of 1:20 (*w*/*v*) using the DMBA assay [22]. These differences could be explained by the use of a lower sample-to-solvent ratio in the present study (1:70, *w*/*v*). Note that, other factors including solar exposure are also known to contribute to significant intra-species PTs variation [44].

Regarding fucoxanthin, in approach 1, the 1_EtOH_70%_Sn extract was revealed to contain 1.34 g of fucoxanthin per 100 g of extract, while none was found in the 1_RTW_Sn extract (Table 1). Contrarily, in approach 2, the 2_EtOH_96%_Sn extract accounted for 0.24 g of fucoxanthin, while only 0.08 g was recovered in the 2_EtOH_70%_Sn extract (Table 1). When comparing both extraction approaches, the results indicate that the 1_EtOH_70%_Sn extract from approach 1 was the purest one. As this extract accounted for 0.07 g of fucoxanthin per 100 g of algae DW, this was revealed to be 27.0% higher than the total fucoxanthin amount recovered together in both the 2_EtOH_96%_Sn and 2_EtOH_70%_Sn extracts (0.05 g/100 g algae DW). The absence of fucoxanthin in the 1_RTW_Sn extract was consistent with previous findings in water extracts from *F. vesiculosus* [45]. These results are in the upper range of fucoxanthin content reported for brown seaweeds [46]. The fucoxanthin content in the 1_EtOH_70%_Sn extract was found to be consistent with that reported in ethanol extracts (0.09 g/100 g algae DW) in the same species [47], as well as when different organic solvents were used, including acetone (0.06 g/100 g algae DW) and methanol (0.08 g/100 g algae DW) [23,38,48]. These findings allow us to suggest that pre-aqueous extraction before using 70% EtOH in the first approach enhanced fucoxanthin recovery, whereas pre-ethanolic extraction (96% EtOH) in the second approach proved detrimental to its recovery. The presence of different physical and chemical barriers in the alga complex matrix has been described to hinder the extraction of fucoxanthin [9].

The 1_RTW_Sn extract, besides PTs, contained 18.6% of carbohydrates. The high abundance of determined Man (59.4 mol%) is possibly due to the occurrence of mannitol, which is known to occur in brown algae [16] and determined via GC-FID alditol acetates to be Man [13,24]. Likewise, the subsequent 1_EtOH_70%_Sn extract was shown to be composed of 10.6% of carbohydrates, with Man accounting for 48.7 mol% (Table 1). For approach 2, a similar trend was observed in the ethanolic soluble extracts, 2_EtOH_96%_Sn and 2_EtOH_70%_Sn, which were found to be composed of 12.0% and 28.8% carbohydrates, with Man comprising 79.7 and 84.9 mol%, respectively (Table 1). The results showed that 2_EtOH_70%_Sn was the purest extract in terms of Man/mannitol content, which amounted to 24.4 g per 100 g of extract. Nevertheless, 1_RTW_Sn was revealed to be the extract richest in Man/mannitol, which accounted for 4.3 g per 100 g of the alga DW, compared to the 3.4 g in 2_EtOH_70%_Sn, the 1.8 g in 2_EtOH_96%_Sn, and the 0.3 g in 1_EtOH_70%_Sn. In fact, the 1_RTW_Sn recovered 66.7% of the initial Man/mannitol content present in *F. vesiculosus* (6.4% DW). In addition, the presence of mannitol in the ethanolic fractions was consistent with previous works, which described it as the main sugar present in ethanol/water (80/20; *v*/*v*) extracts from brown seaweed *Ascophyllum nodosum* [17,18]. In fact, low-molecular-weight sugars like mannitol are generally quite soluble in aqueous ethanol due to their many hydroxyl groups [37,49,50].

Overall, extraction approach 1 recovered 27.0% more total fucoxanthin than did the second one, but 44.2% less of total PTs and 11% less of Man/mannitol. To provide more detail, it can be concluded that approach 1 enabled the obtention of a crude water extract at room temperature with the highest yield containing water-soluble PTs and nearly all the Man/mannitol content, as well as a subsequent 70% EtOH extract containing the highest fucoxanthin content. In turn, approach 2 yielded the richest PTs extract, but demanded more ethanol for mannitol extraction without significant fucoxanthin recovery. Therefore, it seems that approach 1 stands out as a more environmentally friendly and cost-effective strategy, yielding well-balanced extracts containing PTs, Man/mannitol and fucoxanthin. Brown macroalga phenolic- and pigment-rich extracts have been exploited as natural colorants and preservatives in food and foodstuffs [45,46]. Also, it should be noted that mannitol in the food-grade extracts obtained in this study can act as a natural sweetener, which may attenuate the astringency typically associated with PTs to a certain extent.

### 3.3. Recovery of Water Soluble Polysaccharide-Rich Fractions

Water-based extraction solutions are the most used methodology for extracting marine algal polysaccharides, using temperatures between 25 and 90 °C and diluted acidic solutions [3,37]. Aqueous-based extractions are cheap, non-toxic, eco-friendly and provide an easily implemented process for the development of functional foods. Dialysis is a simple and necessary purification step when studying polysaccharides [11,37]. The sulphate ester residues (-SO_3_^−^) that are identified in the dialyzed samples can be inferred to be constituents of the polysaccharides [26].

**Figure 4:**
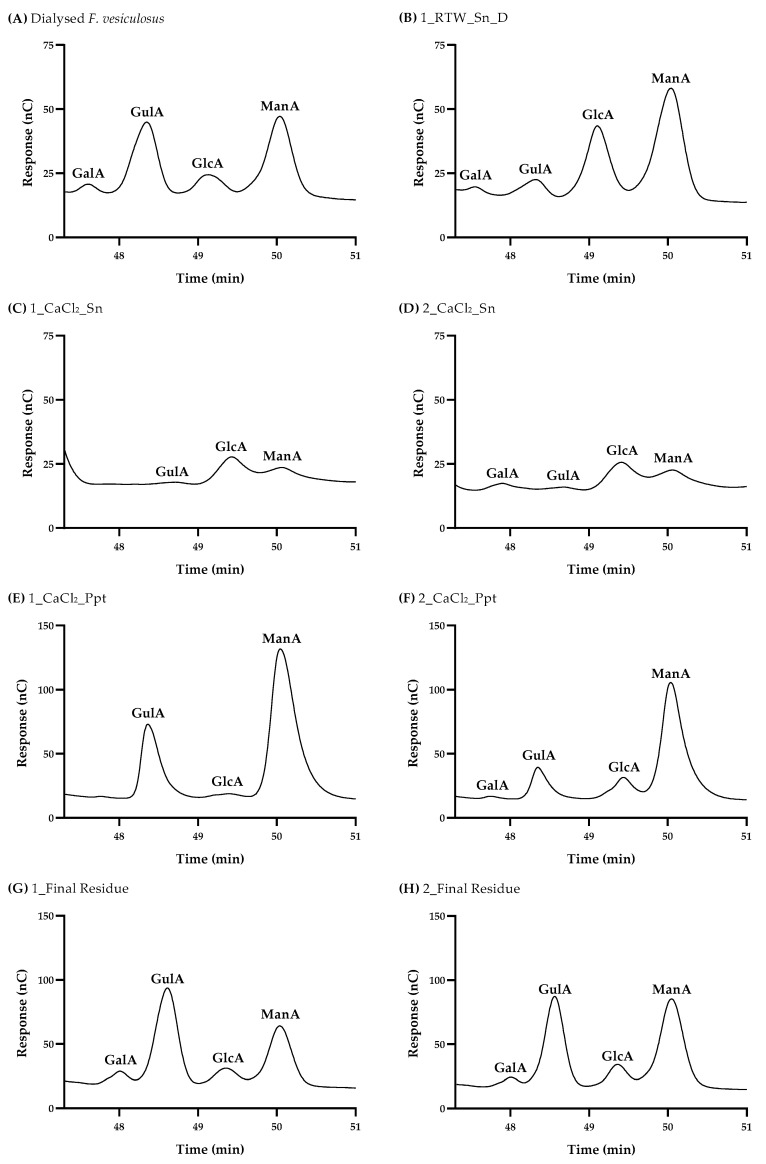
HPAEC-PAD chromatograms of (**A**) dialyzed *F. vesiculosus*, (**B**) 1_RTW_Sn_D, (**C**) 1_CaCl_2__Sn, (**D**) 2_CaCl_2__Sn, (**E**) 1_CaCl_2__Ppt, (**F**) 2_CaCl_2__Ppt, (**G**) 1_Final Residue and (**H**) 2_Final Residue.

In extraction approach 1, the dialyzed water fraction obtained at RT (1_RTW_Sn_D) allowed the recovery of 5.0% of the *F. vesiculosus* polymeric material, which was composed of 38.9% of total carbohydrates (Table 1). This fraction was revealed to be composed mainly of -SO_3_^−^ and Fuc, corresponding to 39.3 and 18.9 mol%, respectively, showing the occurrence of fucoidans, which are commonly found in the cell walls of brown macroalgae [11,12,37,49]. The 1_RTW_Sn_D fraction was also shown to contain Glc (16.8 mol%), indicating the occurrence of laminarans, as well as uronic acids (UA, 16.1 mol%), which could be indicative of the presence of alginates or uronic acids linked to the fucoidan structure, as previously reported [13,37]. This fraction also had minor amounts of Gal, Xyl, Man, Rha and Ara, accounting together for 8.9 mol%, which may also be associated with the fucoidan structure [3,11,12]. Also, minor amounts of PTs were found, corresponding to 0.7 g PGE/100 g extract (Table 1). The total phenolic content was 14.0 g PGE/100 g of extract (Table 1), in agreement with previous studies that indicated a content between 4.5 and 17.6 g PGE/100 g of extract for similar aqueous fractions from *Fucus* spp. [42,51,52,53]. Four distinct uronic acids were identified via high performance anion exchange chromatography (HPAEC-PAD) in the 1_RTW_Sn_D fraction: mannuronic acid (ManA, 46.6 mol%), glucuronic acid (GlcA, 43.8 mol%), guluronic acid (GulA, 5.1 mol%) and galacturonic acid (GalA, 4.5 mol%) (Table 2 and Figure 4B). The higher abundance of ManA and the presence of GulA are indicative of the presence of alginates. The presence of GlcA indicates that this fraction may also contain fucoidans [12,28]. Although less common in *F. vesiculosus*, the presence of GalA is also hypothesized to be associated with the fucoidan structure, aligning with earlier findings [54].

The soluble fraction resulting from the extraction of the insoluble ethanolic residue (1_Residue 2) with hot water followed by precipitation with CaCl_2_ (1_CaCl_2__Sn), used in approach 1, allowed the recovery of 8.3% (*w*/*w*) of the *F. vesiculosus* polymeric material composed of 51.0% of carbohydrates (Table 1). This fraction was shown to mainly contain -SO_3_^−^ (38.8 mol%) and Fuc (37.4 mol%), followed by UA (13.3 mol%), Gal (4.1 mol%), Xyl (3.5 mol%) and minor amounts of Glc, Man, Rha and Ara, characteristic of fucoidans [3]. These results are consistent with previous works on hot water extracts from *F. vesiculosus* [11,12,50,55], as well as from a commercial *F. vesiculosus* fucoidan [26,28]. In addition, the trace amounts of Glc (1.6 mol%) found in 1_CaCl_2__Sn may be linked to the structure of fucoidans, as previously described [12,18], rather than being attributed to the presence of laminarans. The 1_CaCl_2__Sn fraction also contained 3.3% of protein and a total phenolic content of 3.1 g PGE/100 g of extract, consistent with earlier findings [50]. Uronic acids mainly comprised GlcA (71.5 mol%), possibly forming part of the fucoidan structure (Table 2 and Figure 4C). Nevertheless, small proportions of ManA (26.0 mol%) and GulA (2.5 mol%) were also quantified, and these are attributed to co-extracted alginates that were not able to precipitate with CaCl_2_. The alginates recovered in this fraction presented a M/G ratio of 12.56 (Table 2), which is higher (*p* < 0.05) than that of 1_RTW_Sn_D (9.12). This suggests that the first extraction with water was able to solubilize alginates with a higher prevalence of ManA residues.

Briefly, 1_CaCl_2__Sn accounted for 4.2 g of carbohydrates per 100 g of the alga DW, which is 55.8% higher than the amount recovered in 1_RTW_Sn_D (1.9 g). The amount of -SO_3_^−^ (1.5 g) and Fuc (1.6 g) in the 1_CaCl_2__Sn fraction per 100 g of algae was shown to be 56.9% and 77.6% higher compared with the 1_RTW_Sn_D fraction (0.7 g of -SO_3_^−^ and 0.4 g of Fuc). Despite these differences, it was observed that the ratio between -SO_3_^−^ and Fuc in 1_RTW_Sn_D was about two-fold higher than that in 1_CaCl_2__Sn, corresponding to 1.80 and 0.95, respectively, being within the range between 0.5 and 2.5 reported in previous studies [12,56,57]. These results highlight the potential of the developed sequential extraction approach to yield extracts containing fucoidans with potentially different structural characteristics, especially those with a higher degree of sulfation, which is related with stronger biological activities [58]. Besides -SO_3_^−^ and Fuc, the content of Glc in 1_CaCl_2__Sn (0.07 g/100 g algae) was about four-fold lower than that in 1_RTW_Sn_D (0.3 g/100 g algae). This finding emphasizes the potential of water at RT for extracting laminarans, while making the subsequent fractions, i.e., 1_CaCl_2__Sn purest in fucoidans.

Regarding approach 2, the soluble fraction resulting from the extraction of the insoluble ethanolic residue (2_Residue 2) with hot water followed by precipitation with CaCl_2_ (2_CaCl_2__Sn) was able to recover 7.6% (*w*/*w*) of the *F. vesiculosus* polymeric material composed of 44.5% of carbohydrates (Table 1). This fraction was shown to be mainly composed of -SO_3_^−^ (31.4 mol%) and Fuc (25.0 mol%), followed by UA (18.1 mol%) and Glc (13.1 mol%), while minor amounts of Gal, Xyl, Man, Rha and Ara accounted together for 12.4 mol%, characteristic of fucoidans, alginates and laminarans. Earlier studies have described a similar carbohydrate profile in hot water extracts from *F. vesiculosus* samples that had previously undergone ethanolic fractionation [37,50]. Higher levels of protein (9.9%) and phenolic compounds (6.2 g PGE/100 g extract) were observed for the 2_CaCl_2__Sn fraction compared to the approach 1 counterpart. The uronic acid profile of 2_CaCl_2__Sn closely resembles to that of the 1_CaCl_2__Sn fraction from approach 1, thus confirming the presence of co-extracted alginates with a comparable M/G ratio of 12.41 (*p* > 0.05) (Table 2 and Figure 4D).

Furthermore, the 2_CaCl_2__Sn fraction accounted for 3.4 g of carbohydrates per 100 g of the alga DW, which is 20.9% lower than that recovered in 1_CaCl_2__Sn (4.3 g). Specifically, the results indicate that 2_CaCl_2__Sn was only able to recover 0.9 g of -SO_3_^−^ and 0.9 g of Fuc per 100 g of algae, revealing this recovery to be 41.0% and 47.2% lower compared to that of its first approach counterpart, respectively. Despite this, it seems that fucoidans extracted in these two fractions could be similar, as the -SO_3_^−^/Fuc ratio determined for 2_CaCl_2__Sn corresponding to 1.05 is comparable to that observed in 1_CaCl_2__Sn (0.95). The lower recovery of -SO_3_^−^ and Fuc in the 2_CaCl_2__Sn fraction was balanced in part by the enrichment in Glc, which revealed to be 84.7% higher compared to that of its first approach counterpart. In fact, as approach 2 did not include the initial extraction step with water at room temperature, laminarans could only be recovered during hot water extraction. This suggestion aligns with previous findings, which have reported the presence of Glc derived from co-extracted laminarans in CaCl_2_-soluble fractions obtained from *F. vesiculosus* previously submitted to ethanolic fractionation [50].

Overall, it may be concluded that the use of water at RT, instead of 96% ethanol, had a huge impact on the carbohydrate composition present in the subsequently obtained aqueous fractions. Following approach 1, water at RT was able to recover fucoidans with a higher -SO_3_^−^/Fuc ratio and laminarans, whereas the subsequent CaCl_2_-soluble fraction from hot water extraction was the purest in fucoidans, although with a lower -SO_3_^−^/Fuc ratio. On the other hand, following approach 2, it seems that the CaCl_2_-soluble fraction contained fucoidans with a similar -SO_3_^−^/Fuc ratio to that obtained in the approach 1 counterpart, but was also revealed to be the fraction richest in laminarans. Therefore, it can be concluded that approach 1 was able to produce brown macroalga pure polysaccharide fractions, taking advantage of their different solubilities. Laminaran- and fucoidan-rich extracts have demonstrated a positive impact on gut health due to their recognized prebiotic effects [15,59,60,61]. Also, some reports have been exploring them as promising natural anti-oxidant and anti-bacterial agents for improving food products’ shelf-life [46,62], as well as promising hypocholesterolemic agents [13].

### 3.4. Characterization of the Alginate-Rich Fractions

#### 3.4.1. Calcium Precipitates

As hydrocolloids, the most important property of alginates is their ability to form ionic gels in the presence of polyvalent cations, such as Ca^2+^. Based on this, a solution of CaCl_2_ was added to the soluble fraction recovered from hot water extraction, allowing the obtention of precipitates rich in calcium alginates, namely 1_CaCl_2__Ppt and 2_CaCl_2__Ppt (Figure 1).

In the first extraction approach, 4.6% (*w*/*w*) of the *F. vesiculosus* polymeric material was recovered in the 1_CaCl_2__Ppt fraction, containing 75.6% of carbohydrates (Table 1). This fraction was composed mainly of UA, accounting for 90.1 mol%, indicative of the presence of alginates commonly found in brown macroalgae [1,37,40]. Furthermore, 1_CaCl_2__Ppt was also composed of minor amounts of -SO_3_^−^, Fuc, Glc, Man, Gal and Xyl, which together comprised 9.9 mol%, possibly due to a small proportion of co-precipitated fucoidans. On the other hand, the 2_CaCl_2__Ppt fraction from extraction approach 2 was only able to recover 1.8% (*w*/*w*) of the macroalga polymeric material, containing 54.9% of carbohydrates (Table 1). Likewise, the most prominent components in this fraction were UA (73.9 mol%), followed by -SO_3_^−^ (14.5 mol%), Fuc (6.3 mol%) and minor amounts of Glc, Man, Gal and Xyl, together comprising 5.3 mol%. Furthermore, the results indicate that the 1_CaCl_2__Ppt fraction was the purest, as it contained 27.4% more carbohydrates and 40.4% more uronic acids than did the 2_CaCl_2__Ppt fraction. In addition, the amount of carbohydrates in 1_CaCl_2__Ppt accounted for 3.4 g per 100 g of the alga DW, which was revealed to be 70.6% higher than the amounts recovered in 2_CaCl_2__Ppt (1.0 g/100 g algae DW). Similar carbohydrate amounts (71.0% and 62.1%) and UA abundances (72.1 mol% and 93.5 mol%) were reported in previous studies on calcium alginates recovered from hot water extracts of *F. vesiculosus* [49] and *S. latissima* [13], respectively, that also contained co-precipitated fucoidans in their alginate-rich precipitates, with Fuc accounting for 17.5 mol% and 3.8 mol%, respectively. Commercial alginate has also been reported to contain fucoidan in an amount of up to 6.0 mol% Fuc as an impurity [28].

The uronic acid profile determined via HPAEC-PAD for 1_CaCl_2__Ppt revealed to be mainly composed of ManA (70.0 mol%) and GulA (24.8 mol%) with a minor presence of GlcA (5.4 mol%) (Table 2 and Figure 4E). A slightly different profile was observed for 2_CaCl_2__Ppt, with ManA (65.7 mol%) and GlcA (19.3 mol%) as the most abundant uronic acids, followed by GulA (11.9 mol%) and GalA (2.5 mol%) (Table 2 and Figure 4F). The elution of GlcA and GalA together with ManA and GulA corroborates the residual co-precipitation of fucoidans with alginates. The M/G ratio of the alginates recovered in the 1_CaCl_2__Ppt fraction corresponded to 2.91, which revealed to be almost two-fold lower (*p* < 0.05) than that determined for 2_CaCl_2__Ppt, corresponding to 5.50. Lower M/G ratios ranging between 1.09 and 1.44 were reported for calcium alginate fractions recovered from multi-product sequential extractions from *F. vesiculosus* [37] or other brown macroalgae, possibly due to the application of a different quantification method, namely nuclear magnetic resonance spectroscopy [16]. The M/G ratio found for the dialyzed *F. vesiculosus* powder, corresponding to 1.35, was showed to be two-fold and four-fold lower (*p* < 0.05) than that of 1_CaCl_2__Ppt and 2_CaCl_2__Ppt, respectively. This means that the alginates present in the macroalgae are richer in GulA, whereas those extracted with hot water and precipitated with CaCl_2_ are preferentially rich in ManA residues. Alginates with higher GulA levels commonly resulted in stronger and brittle gels useful for food applications, whereas alginates with higher ManA levels resulted in more elastic and flexible gels useful for producing polyelectrolyte complexes and nanoparticles [16,37]. Overall, it can be concluded that using water at RT (approach 1) instead of 96% ethanol (approach 2) may be a simple strategy for modulating the composition of Ca alginate fractions. Therefore, approach 1 seems to be a potential extraction strategy for obtaining purer Ca alginates with a higher prevalence of GulA residues, and consequently with a lower M/G ratio.

#### 3.4.2. Final Residues

After the sequential extraction of *F. vesiculosus* with hot water, the alga residual biomass was generated for both extraction approaches, designated as 1_Final Residue and 2_Final Residue (Figure 1). 1_Final Residue, resulting from approach 1, represented 42.3% (*w*/*w*) of the *F. vesiculosus* material, still containing 12.7 g of carbohydrates per 100 g of the alga DW, which corresponds to 43.2% of the initial carbohydrate content present in the macroalgae (Table 1). This residue was shown to be mainly composed of UA (37.6 mol%), Glc (20.4 mol%), -SO_3_^−^ (17.6 mol%) and Fuc (16.6 mol%). On the other hand, 2_Final Residue, from approach 2, yielded 52.1% (*w*/*w*) of the macroalga material, containing 19.6 g of carbohydrates per 100 g of the alga DW, which represents 66.7% of the initial carbohydrate content present in *F. vesiculosus*. Likewise, the main constituents of this residue were UA (39.3 mol%), -SO_3_^−^ (25.0 mol%), Fuc (20.0 mol%) and Glc (10.3 mol%).

Moreover, 2_Final Residue retained 37.7% more UA (7.7 g/100 g of the alga DW) than did its first approach counterpart (4.8 g). Its HPAEC-PAD profile revealed a higher proportion of ManA (42.2 mol%) than that in 1_Final Residue (28.8 mol%) (Table 2, Figure 4G,H). On the contrary, it presented a lower GulA content (31.2 mol% against 39.9 mol%, respectively). This resulted in a M/G ratio (1.35) that was two-fold higher (*p* > 0.05) than that of 1_Final Residue (0.71). These differences may be explained by the ability of water at RT to extract/purify soluble ManA-richer alginates from *F. vesiculosus*, as carried out in the first approach. This is in accordance with the M/G ratio found for dialyzed *F. vesiculosus* (1.35). In addition to alginates, the high content of -SO_3_^−^ and Fuc per 100 g of the alga DW in both residues means that fucoidans were still present, mainly in 2_Final Residue, which retained 58.3% more -SO_3_^−^ and 46.2% more Fuc than its first approach counterpart.

**Figure 5:**
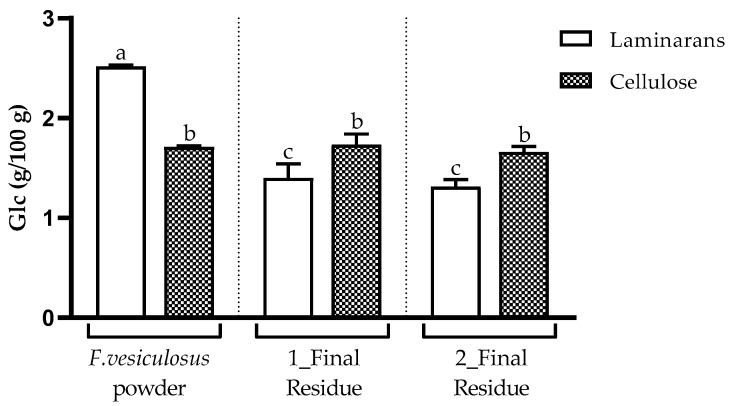
Laminarans (determined as non-cellulosic Glc) and cellulose, expressed as g per 100 g of the alga DW, for the *F. vesiculosus* powder and for its respective final residues from the two extraction approaches. Data are expressed as the means of three replicates ± standard deviation. Different letters represent significant differences (*p* < 0.05).

The content of cellulose present in the *F. vesiculosus* powder was 1.7 g/100 g of the alga DW (Figure 5). This was determined via the difference between the content of Glc released with and without H_2_SO_4_ pre-hydrolysis, as described in the experimental section. The amount of cellulose determined in 1_Final Residue was similar to that in 2_Final Residue (*p* > 0.05), corresponding on average to 1.7 g/100 g of the alga DW. This amount was shown to be consistent with what was estimated for *F. vesiculosus*, because cellulose, due to its water-insoluble character, was expected to remain in the final residues. In addition, the amount of laminarans, determined as non-cellulosic Glc released by acid hydrolysis without H_2_SO_4_ pre-hydrolysis, present in the *F. vesiculosus* powder, was 2.5 g/100 g of the alga DW (Figure 5). The amounts of laminarans were revealed to be similar (*p* > 0.05) between the final residues from the two extraction approaches (1.4 and 1.3 g/100 g of the alga DW, respectively), which means that, on average, 50.0% of the initial laminarans present in the *F. vesiculosus* powder remained in both residues.

Overall, it can be concluded that the initial extraction of the algae with 96% ethanol (approach 2) instead of water at RT (approach 1) can result in final residues with a higher content of alginates and fucoidans, although with a similar content of laminarans and cellulose. This means that both residues still contain a sizeable edible proportion that can be exploited as a source of high-value-added compounds, particularly alginates with a low M/G ratio. In the literature, alginates from the residual biomass of *F. vesiculosus* [11] and other brown macroalgae [16,40] have been recovered with alkaline extractions (e.g., Na_2_CO_3_).

## 4. Conclusions

This work showed the potentiality of endemic seaweeds to be exploited through holistic and cascade processes to produce high-value food products, contributing to a circular and sustainable blue bioeconomy. The initial step of the sequential extractions developed for the brown macroalga *Fucus vesiculosus* has the ability to modulate the composition of the subsequent extracts and/or fractions. The use of water at room temperature (approach 1) instead of 96% ethanol (approach 2) seems to act as a pre-washing step, cleaning water-soluble phenolics, laminarans and ManA-rich alginates. This enabled to obtain, with 70% EtOH, subsequent fractions that were purest in terms of fucoxanthin content. Sequential extraction with hot water allowed to obtain fractions that were purest in terms of fucoidans and alginates with a lower M/G ratio. On the other hand, the use of 96% ethanol in the first extraction step of approach 2, despite recovering the highest amount of phlorotannins, was not successful in extracting fucoxanthin, and less pure subsequent fractions extracted with hot water containing fucoidans, laminarans and alginates with a higher M/G ratio were obtained. Overall, initial extraction with water at room temperature stands out as an environmentally friendly and cost-effective strategy to obtain food-grade extracts rich in specific compounds that could be used as potential ingredients in functional foods.

## Figures and Tables

**Table 1:** Yield (%), total phlorotannins (g PGE/100 g_extract_), total phenolics (g PGE/100 g_extract_), total fucoxanthin (g/100 g_extract_), carbohydrate composition (mol%), total carbohydrates (g/100 g_extract_), total sulphates (g/100 g_extract_) and total protein (g/100 g_extract_) of *Fucus vesiculosus* powder and its respective extracts and/or fractions obtained from the first and the second holistic extraction approach. Data are expressed as the means of three replicates ± standard deviation. nd means not determined. na means not applicable. Extraction yields are calculated in relation to the *F. vesiculosus* dry powder. Indented fractions are derived from the non-indented previous one. * Possibly mannitol, which occurs as mannose, as determined via the methodology used. PGE stands for phloroglucinol equivalents. Sample abbreviations correspond to the sequential extractions represented in Figure 1.

Sample	Yield (%)	Phlorotannins (g PGE/100 g)	Phenolics (g PGE/100 g)	Fucoxanthin (g/100 g)	Carbohydrates (mol%)									Carbohydrates (g/100 g)	Sulphates (g/100 g)	Protein (g/100 g)	
					**Rha**	**Fuc**	**Ara**	**Xyl**	**Man**	**Gal**	**Glc**	**UA**	**SO_3_^−^**				
*Fucus* Powder					0.0	23.5	0.8	2.3	21.7 *	4.0	14.4	33.3	na	29.4 ± 1.7	5.8 ± 0.4	9.6 ± 0.3	
Approach (1)																	
1_RTW_Sn	38.3 ± 3.1	0.36 ± 0.05	2.0 ± 0.03	0.00	0.2	9.5	0.6	1.8	59.4 *	2.3	14.1	12.1	na	18.6 ± 1.0	3.7 ± 0.5	3.3 ± 0.05	
1_RTW_Sn_D	5.0 ± 0.3	0.67 ± 0.04	14.0 ± 0.8	0.00	0.4	18.9	0.3	1.8	1.6	4.8	16.8	16.1	39.3	38.9 ± 2.4	12.9 ± 1.2	nd	
1_EtOH_70%_Sn	5.5 ± 0.1	2.03 ± 0.22	2.5 ± 0.09	1.34 ± 0.14	0.9	1.4	0.0	3.3	48.7 *	35.3	4.9	5.5	na	10.6 ± 0.3	nd	2.4 ± 0.5	
1_CaCl_2__Sn	8.3 ± 0.4	0.10 ± 0.02	3.1 ± 0.5	nd	0.2	37.4	0.2	3.5	0.8	4.1	1.6	13.3	38.8	51.0 ± 2.3	18.1 ± 0.3	3.3 ± 0.8	
1_CaCl_2__Ppt	4.6 ± 0.4	0.00	0.4 ± 0.1	nd	0.2	2.7	0.0	0.4	0.9	0.3	0.8	90.1	4.6	75.6 ± 6.9	1.8 ± 0.3	1.7 ± 0.1	
1_Final Residue	42.3 ± 0.3	nd	nd	nd	0.0	16.6	0.8	2.6	1.8	2.5	20.4	37.6	17.6	30.0 ± 1.2	3.4 ± 0.6	17.9 ± 0.4	
Approach (2)																	
2_EtOH_96%_Sn	18.0 ± 0.5	2.38 ± 0.34	8.4 ± 0.8	0.24 ± 0.02	0.0	1.3	0.0	2.7	79.7 *	10.6	5.7	0.0	na	12.0 ± 1.6	nd	3.5 ± 0.1	
2_EtOH_70%_Sn	14.0 ± 0.3	0.63 ± 0.03	1.6 ± 0.2	0.08 ± 0.01	0.3	0.5	0.5	0.6	84.9 *	2.0	7.3	5.1	na	28.8 ± 2.7	nd	nd	
2_CaCl_2__Sn	7.6 ± 4.0	0.11 ± 0.02	6.2 ± 0.5	nd	0.7	25.0	0.2	3.3	1.8	6.4	13.1	18.1	31.4	44.5 ± 1.6	11.1 ± 1.6	9.9 ± 0.5	
2_CaCl_2__Ppt	1.8 ± 0.1	0.00	1.8 ± 0.2	nd	0.2	6.3	0.2	1.5	1.7	0.5	1.7	73.9	14.5	54.9 ± 7.3	4.7 ± 0.1	5.8 ± 0.5	
2_Final Residue	52.1 ± 0.4	nd	nd	nd	0.2	20.0	0.3	1.9	1.0	2.0	10.3	39.3	25.0	37.7 ± 4.0	6.6 ± 0.7	12.7 ± 1.0	

**Table 2:** Uronic acid (UA) profile (mol%) and content (g/100 g of sample) and mannuronic acid to guluronic acid ratio (M/G) of dialyzed *F. vesiculosus* and its respective fractions obtained from the first and second extraction approach (Figure 1).

Sample	Uronic Acids (mol%)				Total UA (g/100 g)	M/G Ratio
	**GalA**	**GulA**	**GlcA**	**ManA**		
Dialysed *F. vesiculosus*	10.9	30.3	17.9	40.8	19.2 ± 2.5	1.35 ± 0.02 ^a^
Approach (1)						
1_RTW_Sn_D	4.5	5.1	43.8	46.6	11.3 ± 1.4	9.12 ± 0.1 ^b^
1_CaCl_2__Sn	0.0	2.5	71.5	26.0	12.6 ± 1.4	12.56 ± 0.5 ^c^
1_CaCl_2__Ppt	0.0	24.8	5.4	70.0	71.4 ± 7.6	2.91 ± 0.09 ^d^
1_Final Residue	18.7	39.9	13.0	28.3	14.6 ± 0.2	0.71 ± 0.02 ^a^
Approach (2)						
2_CaCl_2__Sn	20.3	2.9	47.1	29.8	13.0 ± 0.3	12.41 ± 1.9 ^c^
2_CaCl_2__Ppt	3.1	11.9	19.3	65.7	51.7 ± 3.6	5.50 ± 0.09 ^e^
2_Final Residue	12.4	31.2	14.2	42.2	23.4 ± 1.7	1.35 ± 0.06 ^a^

Data are expressed as the means of three replicates ± standard deviation. Different letters represent significant differences (*p* < 0.05).

## Data Availability

The data that support the findings of this study are available within this article.
